# Black yeasts in hypersaline conditions

**DOI:** 10.1007/s00253-024-13052-2

**Published:** 2024-03-05

**Authors:** Cene Gostinčar, Nina Gunde-Cimerman

**Affiliations:** Biotechnical Faculty, Department of Biology, University of Ljubljana, Jamnikarjeva 101, 1000 Ljubljana, Slovenia

**Keywords:** Extremophile, Halotolerant, Black yeast, Adaptation, Hypersaline

## Abstract

**Abstract:**

Extremotolerant and extremophilic fungi are an important part of microbial communities that thrive in extreme environments. Among them, the black yeasts are particularly adaptable. They use their melanized cell walls and versatile morphology, as well as a complex set of molecular adaptations, to survive in conditions that are lethal to most other species. In contrast to extremophilic bacteria and archaea, these fungi are typically extremotolerant rather than extremophilic and exhibit an unusually wide ecological amplitude. Some extremely halotolerant black yeasts can grow in near-saturated NaCl solutions, but can also grow on normal mycological media. They adapt to the low water activity caused by high salt concentrations by sensing their environment, balancing osmotic pressure by accumulating compatible solutes, removing toxic salt ions from the cell using membrane transporters, altering membrane composition and remodelling the highly melanized cell wall. As protection against extreme conditions, halotolerant black yeasts also develop different morphologies, from yeast-like to meristematic. Genomic studies of black yeasts have revealed a variety of reproductive strategies, from clonality to intense recombination and the formation of stable hybrids. Although a comprehensive understanding of the ecological role and molecular adaptations of halotolerant black yeasts remains elusive and the application of many experimental methods is challenging due to their slow growth and recalcitrant cell walls, much progress has been made in deciphering their halotolerance. Advances in molecular tools and genomics are once again accelerating the research of black yeasts, promising further insights into their survival strategies and the molecular basis of their adaptations.

**Key points:**

• *Black yeasts show remarkable adaptability to environmental stress*

• *Black yeasts are part of microbial communities in hypersaline environments*

• *Halotolerant black yeasts utilise various molecular and morphological adaptations*

## Introduction

Life in extreme environments is governed by physico-chemical parameters that are close to the limits of life. Although they are inhospitable for most species, they are inhabited by vibrant communities of well-adapted extremotolerant and extremophilic organisms. Historically, extreme environments have been thought of as the domain of prokaryotes, but in recent decades, they have been shown to harbour much greater diversity. Fungi are now recognised as an integral part of extreme microbial communities (Gostinčar et al. 2023).

**Fig. 1:**
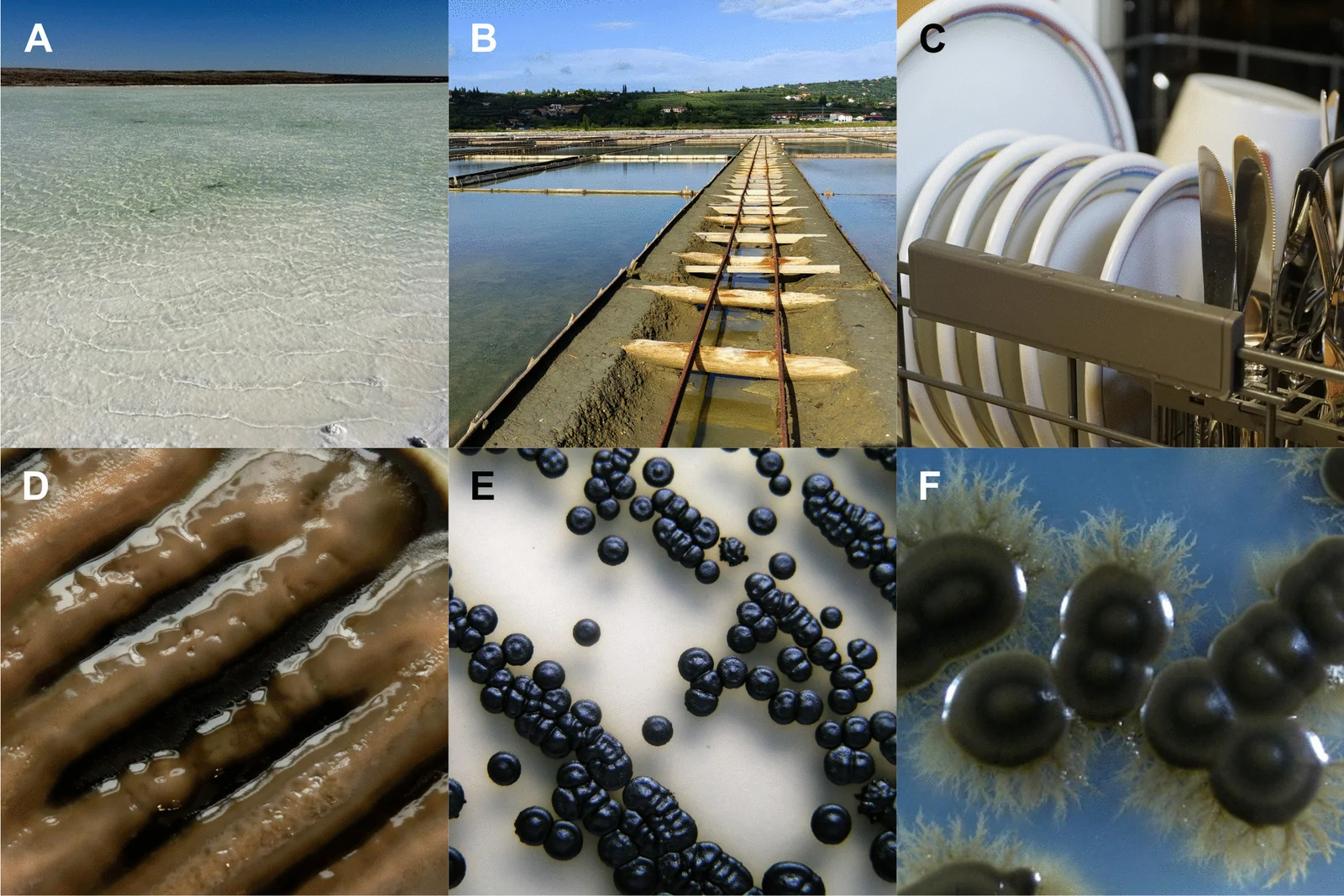
Selected environments in which halotolerant black fungi are commonly found (**A**–**C**) and the typical representative species of each environment below them (**D**–**F**). (**A**) A hypersaline lake in the Kalahari Desert, South Africa. (**B**) Evaporation ponds of solar saltern Sečovlje, Slovenia. (**C**) Household dishwasher. (**D**) *Aureobasidium melanogenum*. (**E**) *Hortaea werneckii*. (**F**) *Exophiala dermatitidis*

While bacteria and archaea from extreme environments often struggle to survive in temperate conditions, fungi typically have a greater ecological amplitude – they are extremotolerant and not extremophilic (Gostinčar et al. 2022b). However, although most extremotolerant fungi can adapt to temperate conditions in vitro, they do not thrive under such conditions in nature, as they may not be able to compete with mesophilic species. Consequently, many extremotolerant fungal species show clear habitat specialisation and only thrive in certain extreme conditions such as high salinity (Fig. 1) (Gunde-Cimerman et al. 2018).

Hypersaline environments are increasingly scrutinised by microbiologists due to the effects of climate change and the discovery of thriving halotolerant and halophilic microbial communities (Gostinčar and Gunde-Cimerman 2023). As with other extreme environments, they were initially thought to be inhabited mainly by archaea and bacteria. Although salt tolerance in eukaryotes was known from research on the alga *Dunaliella salina* (Gunde-Cimerman et al. 2018) and fungal contamination on salted foods, eukaryotic fungal cells were long thought to be too complex to successfully adapt to extreme concentrations of extracellular salt. Any isolates were dismissed as transient contaminants, and little consideration was given to the possibility of active fungal growth at high salinity, let alone to active sampling for halotolerant or halophilic fungi (Gunde-Cimerman et al. 2000, 2009). Only after repeated isolation of the same halotolerant species from hypersaline habitats in different parts of the world did it become clear that extremophilic fungi, especially black yeasts, are a crucial component of these hypersaline environments (Fig. 1) (Gunde-Cimerman et al. 2000).

Black yeasts are a phylogenetically diverse group of melanized fungi, which exhibit a remarkable range of cellular adaptations and distinct morphologies that enable them to respond to various environmental stresses (Tiquia-Arashiro and Grube 2019). Interestingly, the extremotolerance that allows survival in harsh environments may overlap with mechanisms of persistence in a human host, and certain black yeasts can cause opportunistic infections (Gostinčar et al. 2018). In this review, we focus on the biodiversity and distinctive features of halotolerant and extremely halotolerant black yeasts – those that can grow at salt concentrations above 10% NaCl (w/v) (Gunde-Cimerman et al. 2018). These species primarily colonise hypersaline, halite-based aquatic environments and occasionally other environments with low water activity, such as bitterns (Zajc et al. 2014), ice (Gunde-Cimerman et al. 2003; Turchetti et al. 2013) and surfaces exposed to desiccation (Coleine et al. 2022).

## Hypersaline and other environments with low water activity

Water is not only a molecule necessary for life as we know it but sustains living organisms only within a narrow range of water availability, expressed as water activity (*a*_*w*_). Water activity is defined as the effective water content expressed in mole fraction, where pure water has an *a*_*w*_ value of 1.0. The presence of solutes lowers water activity to values below 1.0 and impairs microbial growth by creating osmotic pressure, while some solutes are also directly toxic to cells (Gunde-Cimerman et al. 2018). Biologically available water in nature can therefore be limited by high concentrations of salt or other osmolytes, by freezing and by aridity (Gostinčar et al. 2023). Microorganisms, including some black yeasts, can only survive under such conditions due to osmoregulation and other specialised adaptations (Gunde-Cimerman et al. 2018). The overlap of these adaptations between different conditions (e.g. hypersaline water and glacier ice) is believed to be considerable (Gostinčar et al. 2023), but the extent of independent vs. overlapping adaptations to each of these conditions remains to be determined.

In nature, the most conspicuous hypersaline habitats are saline waters or salt flats with thalassohaline or athalassohaline characteristics. Thalassohaline waters resemble concentrated seawater and are dominated by halite (NaCl). In athalassohaline waters, the ionic composition is determined by the local geology, which can lead to high concentrations of cations such as Mg^2+^, Ca^2+^, K^+^ and Li^+^ (Gostinčar and Gunde-Cimerman 2023). While the global black yeast diversity and their occurrence in thalassohaline brines is relatively well known, there is little information on their occurrence in athalassohaline waters and the effects of chaotropic salts (e.g. MgCl_2_) on their growth (Zajc et al. 2014). Chaotropic salts, unlike cosmotropic salts, disrupt the hydrogen bonding network of water molecules around proteins, reducing structure and order in water and decreasing water activity (Zajc et al. 2014). Black yeasts have been isolated from hypersaline environments around the world, from saltern brines (Gunde-Cimerman et al. 2000; Zalar et al. 2005; Gunde-Cimerman and Zalar 2014; Chung et al. 2019), magnesium-rich bitterns (Zajc et al. 2014), athalassohaline hypersaline waters of the Kalahari inland salterns in South Africa (our unpublished data), hypersaline industrial effluents (Zeng et al. 2021), cold hypersaline lakes in Antarctica (Ogaki et al. 2019) and cryopegs in Siberian permafrost (Gilichinsky et al. 2005). Less obvious black yeast habitats with at least temporary low water activity are glacial ice in polar regions (Perini et al. 2019a, 2019b) and indoor surfaces in kitchens, bathrooms and some household appliances (Zalar et al. 2011).

## Biodiversity of black yeasts in hypersaline environments

Black yeasts are a polyphyletic group of species from the orders *Chaetothyriales*, *Capnodiales* and *Dothideales*. They are known for their adaptability to extreme environmental conditions such as high salinity, low water activity, extreme temperatures and pH values as well as exposure to UV and ionising radiation (Kogej et al. 2006a; Sterflinger 2006).

The black yeasts found in hypersaline waters can be divided into transient taxa, which are strongly influenced by local conditions, and members of resident core communities, which occur in most or all similar environments worldwide. In addition, halotolerant black yeasts can be habitat specialists, occurring only or predominantly in (sometimes very specific) hypersaline environments, and habitat generalists, thriving in both hypersaline and temperate environments. The core species in hypersaline waters include the specialists *Hortaea werneckii*, *Neophaeotheca triangularis* (previously *Phaeotheca triangularis*) (Abdollahzadeh et al. 2020) and *Trimmatostroma salinum* (Butinar et al. 2005), as well as the generalists *Aureobasidium pullulans* and *Aureobasidium melanogenum* (Zalar et al. 1999a, 1999b, 1999c; Butinar et al. 2005; Zajc et al. 2017; Chung et al. 2019).

*Hortaea werneckii* serves as a model organism for eukaryotic halotolerance and is a prime example of a fungus that thrives in halite-based hypersaline environments. This species can grow in near-saturated NaCl solutions and also thrives in normal non-saline mycological media, with a growth optimum between 5 and 10% NaCl. Such an extremotolerant phenotype with a broad ecological amplitude is typical for black yeasts. *H. werneckii* is found worldwide in thalassohaline and hypersaline waters, but also in other environments with low water activity, including magnesium-rich bitterns and cold environments such as deep-sea water and glacial ice. It can grow on wood and secrete cellulases under hypersaline conditions, appears on salted foods and even causes *tinea nigra*, a superficial infection of salty human skin (Gunde-Cimerman et al. 2000, 2018; Butinar et al. 2005; Gunde-Cimerman and Plemenitaš 2006; Gostinčar and Gunde-Cimerman 2023). The extreme halotolerance of *H. werneckii* is associated with an increased number and diversity of secondary metabolites at high NaCl concentrations (our unpublished data). This is a rare exception among fungi to the general rule of reduced synthesis of secondary metabolites at low water activity.

*Trimmatostroma salinum* usually grows on wood (together with *H. werneckii*) immersed in brine. Wood also appears to serve as a dispersal vector. The halotolerant *T. salinum* can survive and multiply at (optimally) 20% NaCl (w/v) (Zalar et al. 1999c, 2005; Gunde-Cimerman et al. 2000).

The halophilic *Neophaeotheca triangularis*, originally described as a new species found in an air-conditioning humidifier, has a narrow ecological range and thrives in environments with NaCl concentrations of 22 to 28% (w/v), but not in saturated solutions (Abdollahzadeh et al. 2020). It is frequently isolated from microbial pellicula that form on the surface of hypersaline water, as well as from saltern microbial mats, suggesting that brine-covered adhesive surfaces are its primary habitat (de Hoog et al. 1997; Butinar et al. 2005).

*Aureobasidium pullulans* is an extremotolerant generalist, characterised by remarkable adaptability rather than extreme specialisation. It is able to tolerate various extreme conditions, from hypersaline (up to 17% NaCl; w/v) to acidic, basic, cold and oligotrophic. It is nutritionally versatile and successful at outcompeting other species. It is found in an exceptionally wide range of habitats from salterns to plant surfaces, house dust and polar glaciers. Like some other generalists, *A. pullulans* has many biotechnological applications. *Aureobasidium melanogenum*, a closely related species, also exhibits varying degrees of extremotolerance, but has a higher temperature optimum and can grow at 37 °C. This characteristic is crucial for its ability to cause opportunistic infections in humans. *A. melanogenum* is frequently isolated from aquatic environments, from non-saline tap water to the hypersaline water of an inland saltern in the Kalahari Desert (South Africa) (our unpublished data). The related *Aureobasidium subglaciale*, which is able to grow at 10% NaCl (w/v), is a specialised species mainly found in Arctic glaciers (Gostinčar and Gunde-Cimerman 2023).

Some of the transient and sporadic halotolerant species found in hypersaline environments are *Cadophora luteo-olivacea, Exophiala dermatitidis, Pseudotaeniolina globosa*, *Salinomyces thailandicus, Zalaria alba* and *Zalaria obscura*. They are generally able to grow up to 15% NaCl (w/v) and are found in a variety of habitats.

*Cadophora luteo-olivacea* has a bipolar distribution in very cold and/or saline environments such as polar soils, freshwater lakes and marine sediments, but also occurs as an endophyte in various green plants and mosses in both high latitudes and temperate-tropical areas. It is able to initiate nutrient cycles, particularly through the soft-rot wood decomposition, which is typical of marine ascomycetes (Hirose et al. 2013; Nagano et al. 2016; Rämä et al. 2017).

The polyextremophilic *Exophiala dermatitidis* is known for its adaptability to various oligotrophic environments, ranging from artificial surfaces to glaciers, and for its tolerance to a wide range of temperatures, pH values and salinities. *E. dermatitidis* has also been associated with neurotropic and other infections in humans, particularly in immunocompromised individuals (Branda et al. 2010; Novak Babič et al. 2018).

*Pseudotaeniolina globosa* is a rock-dwelling fungus usually associated with natural rocky substrates. It is commonly isolated from exposed surfaces such as desert rocks, outdoor statues, leathery plant foliage and hypersaline coastal ponds. It is highly halo- and thermotolerant and grows optimally at 1.5% NaCl and up to 30% NaCl (w/v) and at a broad range of temperatures from 4 to 37 °C (Ortiz et al. 2014; Rizk et al. 2021).

The halotolerant *Salinomyces polonicus*, closely related to *Hortaea werneckii* (Crous et al. 2009; Czachura et al. 2021), can be isolated from sandstone saturated with seawater and can grow up to 15% NaCl.

The halotolerant *Zalaria obscura* and *Zalaria alba*, which can both grow up to 20% NaCl (w/v), are distributed worldwide and occur on various substrates, including wood, soil, dust, sediments, subglacial ice and cultural heritage (Humphries et al. 2017; Sabatini et al. 2020).

## Molecular adaptations to hypersaline conditions

**Fig. 2:**
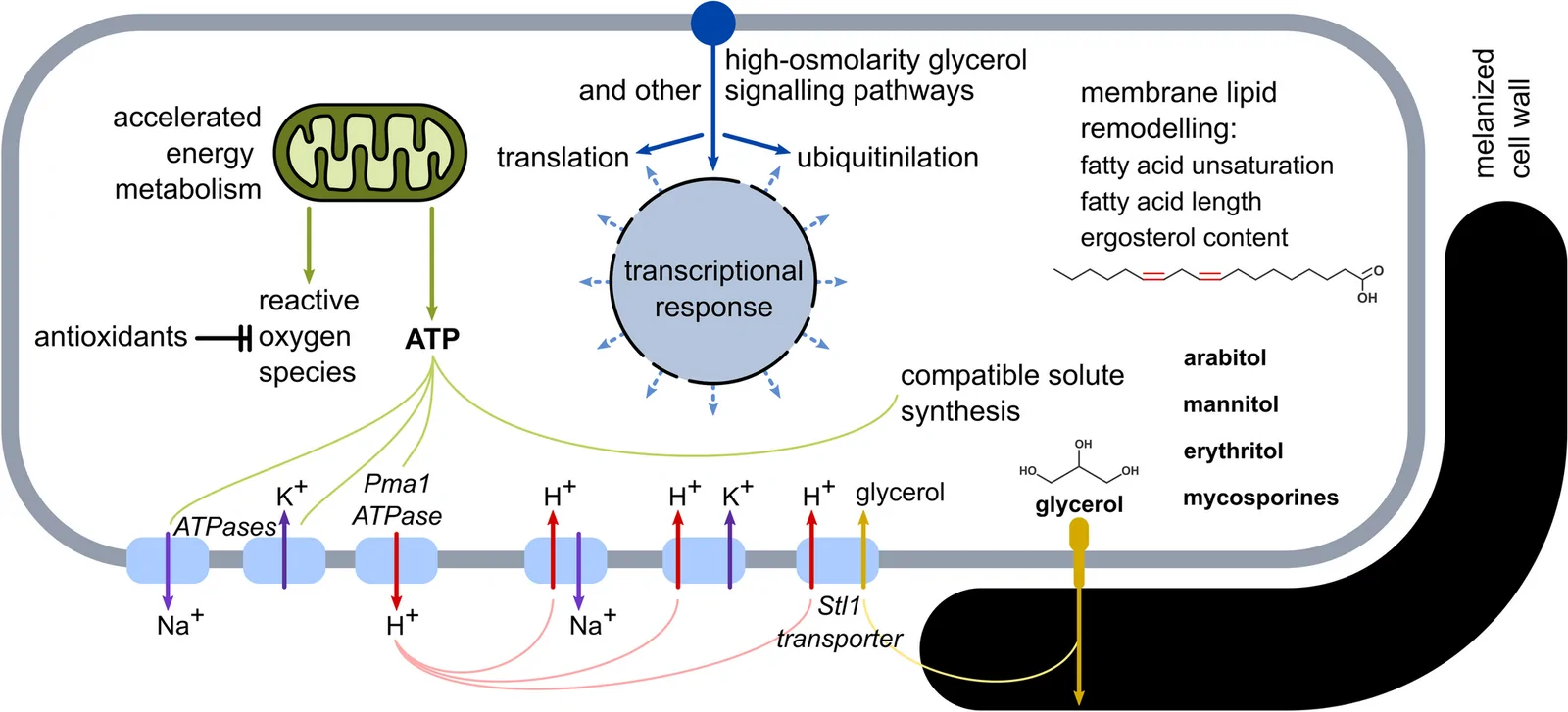
Major adaptations of black yeasts to growth at high salinity. The cells sense the osmolarity of the medium by membrane sensors and convey the information through the high-osmolarity glycerol and other signalling pathways, triggering a complex network of responses. The energy metabolism of the cell is accelerated to power the increased energy expenditure used for the synthesis of compatible solutes, membrane transporters and other adaptations. The resulting increase in oxidative stress is countered by enzymatic and non-enzymatic antioxidants. Accumulation of compatible solutes balances the osmotic pressure, while membrane transporters powered by the transmembrane proton gradient or ATP maintain physiological intracellular concentrations of Na^+^, K^+^ and other ions as well as retrieve compatible solutes that leak from the cell. Cellular membranes and the melanized cell wall are remodelled to provide structural stability, maintain appropriate membrane fluidity and reduce the leakage of compatible solutes

Extremotolerant fungi have evolved to cope with the harsh challenges posed by high-salinity environments. Their remarkable resilience is based on an intricate series of molecular adaptations (Fig. 2) that form a complex halotolerant phenotype, which is only partially understood (Gostinčar and Gunde-Cimerman 2023). Much of the research on halotolerance in fungi has been conducted on relatively salt-sensitive model organisms, mainly *Saccharomyces cerevisiae*, and on cells exposed to sudden changes in salinity (mainly NaCl, e.g. (Chasman et al. 2014)) or non-osmotic solutes (such as sorbitol, e.g. (Gasch et al. 2000)). Although they provide valuable data on the responses of salt-sensitive cells to salinity shocks, neither approach is optimal for understanding the survival of halotolerant fungi in natural environments, where salinity typically increases much more slowly (Gostinčar and Gunde-Cimerman 2023). To remedy this, halotolerant and halophilic fungi and constant high salinity have been proposed as alternative research models, including the black yeasts *H. werneckii* (Gunde-Cimerman et al. 2009) and *A. pullulans* (Gunde-Cimerman and Zalar 2014).

Studies on halotolerant black yeasts have confirmed some of the halotolerance mechanisms already known from mesophiles, albeit sometimes with altered functionality, and identified some additional mechanisms. For example, while the high-osmolarity glycerol (HOG) signal transduction pathway is the general pathway that responds to osmotic (and other) stress in fungi (Hohmann et al. 2007), phosphorylation of the central mitogen-activated protein kinase Hog1 at high NaCl concentration is permanent in *H. werneckii* and only transient in other fungi.

One of the main responses triggered by the HOG signalling pathway is the accumulation of compatible solutes, mainly glycerol, to balance osmotic pressure and prevent loss of turgor and dehydration of the cytosol (Petelenz-Kurdziel et al. 2011). During hyperosmotic shock, this is achieved by the upregulation of glycerol-3-phosphate dehydrogenase (Gpd1), the rate-limiting enzyme in the glycerol synthesis pathway, as shown in *H. werneckii* (Kejžar et al. 2015) and *A. pullulans* (Turk and Gostinčar 2018). However, at a constant high salinity, it seems to be more important to prevent or counteract its constant leakage from the cell (Gostinčar and Gunde-Cimerman 2023). This is easily explained – synthesising large amounts of compatible solutes is one of the major energetic costs at high salinity (Oren 1999). Glycerol can be transported back into cells by Stl1 and its homologues, members of the Major Facilitator Superfamily (Ferreira et al. 2005). Transcription of the corresponding genes increases under hypersaline conditions, and this is one of the most consistent fungal responses to salt, which has also been observed in *H. werneckii* (Kejžar et al. 2015) and *A. pullulans* (Turk and Gostinčar 2018). In the latter, multiplication of the gene was also observed, resulting in more than ten putative copies (Turk and Gostinčar 2018). The reduction of glycerol leakage from the cell has also been proposed as a strategy for halotolerant black yeasts, either by altering the membrane composition (Gostinčar and Gunde-Cimerman 2023) or by impermeability of the cell wall due to melanin, as in the case of *H. werneckii* (Kogej et al. 2007).

In addition to glycerol, black yeasts also accumulate other compatible solutes. In *H. werneckii*, for example, arabitol, mannitol and erythritol were found (Kogej et al. 2007). These compounds require more resources for their production but are also less prone to leak out of the cells, which could explain their preferential accumulation in the stationary growth phase. Other compounds that could act as accessory compatible solutes in *H. werneckii* are mycosporines and mycosporine-like amino acids (Kogej et al. 2006b). All this shows that the diversity of compatible solutes in halotolerant black yeasts is much greater than often assumed. This opens up opportunities for biotechnology, as many of the compounds listed are used in the food, cosmetics and other industries. In addition, each of these compounds may have its own advantages and disadvantages for the cell and provide protection against other types of stress beyond simply balancing osmotic pressure (Grube et al. 2013). Some can act as non-enzymatic scavengers of reactive oxygen species (ROS) (Sharma et al. 2017; Gostinčar and Gunde-Cimerman 2023). Oxidative stress increases under suboptimal growth conditions, also at high salinity, where the increased energy demand combined with imbalances in electron transport chains leads to the formation of highly reactive metabolic by-products that limit the growth of even otherwise well-adapted halotolerant species such as *H. werneckii* (Petrovič 2006; Gostinčar and Gunde-Cimerman 2018).

In addition to the risk of cell dehydration due to differences in osmotic pressure, hypersaline conditions jeopardise the cell through an intracellular accumulation of toxic concentrations of inorganic ions. In thalassohaline environments, Na^+^ is the main problem, but depending on the environment, Mg^2+^, Li^+^ and other ions can be even more detrimental to survival (Plemenitaš et al. 2014). Halotolerant black yeasts have a large variety and number of alkali metal cation membrane transporters to combat ionic imbalances. The large functional redundancy of transporter genes (due to gene duplication and the coexistence of unrelated but functionally similar genes) is thought to play an important role in halotolerance, but it does not correlate perfectly with it. For example, *H. werneckii* contains a large number of transporter genes, especially for Na^+^ and K^+^ (Lenassi et al. 2013), but in *A. pullulans*, the diversity and number of these genes are even greater, although this species is significantly less halotolerant (Gostinčar et al. 2014).

All this leads to an important but often neglected conclusion: that the intracellular environment of halotolerant black yeasts differs significantly from their extracellular environment. This is not the case for all extremes – In the cell of barophiles, there is a high pressure and in psychrophiles a low temperature. In halotolerant fungi, on the other hand, most of the cellular machinery is never exposed to extreme salt concentrations and is not adapted to them (Plemenitaš et al. 2014). The two exceptions are the plasma membrane and the cell wall, and both respond to hypersaline conditions. The composition of the membrane, especially the saturation and length of fatty acids and the amount of sterols are altered to minimise fluctuations in membrane fluidity (Turk et al. 2004). In *H. werneckii* and *A. pullulans*, at least some of these changes are a consequence of the altered expression of genes involved in ergosterol synthesis and the desaturation and elongation of fatty acids (Gostinčar et al. 2008, 2009). The cell wall of halotolerant black yeasts is often highly melanized, which gives these species their characteristic appearance. Melanin is deposited in the thick cell walls or in the outer layer of exopolysaccharides and influences the ability of black yeasts to form biofilms (Kogej et al. 2004, 2006a, 2007; Sterflinger 2006). The melanin layer helps to maintain the integrity of the cell wall. It is also thought to render the cell wall impermeable and reduce glycerol leakage at high salinity (Kogej et al. 2007). Interestingly, solid-state nuclear magnetic resonance (NMR) spectroscopy showed that the cell walls of *H. werneckii* contain only small amounts of chitin and chitosan (our unpublished data). The involvement of the cell wall in halotolerance is also emphasised by the report that during the long-term growth of *H. werneckii* at high salinity, the few genes associated with changes in the genome are related to the cell wall (Gostinčar et al. 2021).

All these adaptations are not without cost. Large amounts of energy are required to maintain the cellular machinery that provides protection from hypersaline conditions, including the continuous synthesis and transport of compatible solutes and the transport of salt ions (Oren 1999). Therefore, at high salinity, *H. werneckii* upregulates genes of the glycolytic pathway, tricarboxylic acid cycle, pentose phosphate pathway and mitochondrial biogenesis (Vaupotič and Plemenitaš 2007). As mentioned above, this can lead to increased oxidative stress, especially near the maximum salinity growth limit.

Despite the complexity of the halotolerant phenotype, some rare cases of single-gene effects on halotolerance have been described. Expression of 3’-phosphoadenosine-5’-phosphatases from *H. werneckii* and *A. pullulans*, or even just an insertion of a 21-amino acid long region of these enzymes in a recipient homologue, has been shown to increase halotolerance in *S. cerevisiae* and *Arabidopsis thaliana* (Vaupotič et al. 2007; Gašparič et al. 2013). But such examples are rare. A comprehensive explanation of fungal halotolerance remains elusive and may require new insights into cellular functions, such as the recent observation of the relationship between the condensation of intrinsically disordered proteins and the amount of free water in the cytoplasm (Watson et al. 2023). At the level of a single protein, the non-canonical sequence of the intrinsically disordered carboxy-terminal domain of RNA polymerase II from *A. pullulans* and *H. werneckii* affects the ability of this enzyme to undergo phase separation in vitro and to localise in vivo (Palumbo et al. 2022), with as yet unknown effects on the transcriptional machinery of these two black yeasts.

## Unusual morphology of halotolerant black yeasts

Molecular adaptations to hypersaline conditions are often accompanied by changes in morphology. While filamentous growth facilitates the exchange of water, nutrient absorption and solutes between cells, this connectivity also makes the cells susceptible to water loss due to the high surface area-to-volume ratio of hyphae, making them more susceptible to water loss (Sterflinger 1998; Gow and Lenardon 2023). In contrast, yeast cells function as individual units surrounded by the protection of the cell wall. But the morphological changes of black yeasts can go far beyond the simple yeast-hyphae dichotomy (Grube et al. 2013). Budding and fission of yeast cells, formation of lateral conidia on hyphae, endoconidiation, meristematic growth and other types of growth, all with varying degrees of melanisation, can be observed and sometimes occur simultaneously in the same culture (Zalar et al. 2008; Slepecky and Starmer 2009; Egidi et al. 2014; Mitchison-Field et al. 2019). This polymorphism plays a crucial role in the adaptation of black yeasts to various environments, from terrestrial to aquatic, including hypersaline habitats.

The high energy requirement for growth under hypersaline conditions, where nutrients are not always abundant, often leads to slow growth (Gostinčar et al. 2022b). Among the morphologies observed in black yeasts, slow-growing clumps of densely packed cells are most commonly associated with extremotolerance. Such meristematic clumps minimise the surface-to-volume ratio and protect the cells inside the clump (Wollenzien et al. 1995; Selbmann et al. 2013). This is particularly important in hypersaline environments where, as mentioned above, the cell surface is the point of contact between the hypersaline medium and protected intracellular space. Meristematic hyphae can also act as exogenous dormancy structures that protect the upper cells in the colony from direct exposure to high salinity (Kogej et al. 2006a; Harris 2011). The mechanisms underlying polymorphic growth are largely unknown but may be triggered by calcium signalling. In *E. dermatitidis*, polymorphism and meristematic clumps are calcium ion dependent (Karuppayil and Szaniszlo 1997).

If a particular morphology is suited to a particular set of environmental conditions, it is difficult to explain why some species, notably *Aureobasidium* spp., sometimes develop a range of morphologies in a single, uniform laboratory medium. This diversity can be seen as a form of bet hedging that contributes to survival in diverse and changing environments (Hewitt et al. 2016), particularly in ecological generalists such as *A. pullulans*, and to a lesser extent in specialists such as the (less polymorphic) *H. werneckii*.

## Genomics as the future of black yeast research

Black yeasts isolated from hypersaline environments are, in many ways, a natural choice for studies of eukaryotic halotolerance. Many of them can be maintained in a predominantly yeast-like state and are therefore easier to manipulate than non-yeast fungi such as the halophilic ascomycete *Aspergillus sydowii* (Pérez-Llano et al. 2020) or the halophilic basidiomycete *Wallemia ichthyophaga* (Zajc et al. 2013). Their halotolerant physiology also makes black yeasts more suitable than relatively salt-sensitive models such as *S. cerevisiae*. Nevertheless, the study of halotolerant black yeasts has long been hampered by the lack of effective molecular tools. Due to their thick cell walls and other (often unknown) factors, the methods used with conventional model fungi must be heavily modified if they can be used at all (Gostinčar and Gunde-Cimerman 2023).

The genome sequencing of *H. werneckii* (Lenassi et al. 2013), *A. pullulans* (Gostinčar et al. 2014), *E. dermatitidis* (Chen et al. 2014) and other black yeasts was an important step that facilitated the study of their extremotolerance. Explaining a phenotype as complex as halotolerance through genomic analyses is a major challenge, but these efforts nevertheless yielded several discoveries, from the multiplication of genes encoding inorganic ion transporters in *A. pullulans* and *H. werneckii* to the unusual genome architecture of *H. werneckii* (Lenassi et al. 2013; Gostinčar et al. 2014). Later, population genomics was used to confirm that *A. pullulans* is a true generalist that can inhabit a variety of environments without cryptic specialisation and even with an unusually high level of recombination (Gostinčar et al. 2019). In contrast, *H. werneckii* has been shown to be strictly clonal, with approximately two-thirds of wild strains being highly heterozygous diploid hybrids. This phenomenon was described as stable parasexuality and is proposed as a means to avoid the recombination load and energy expenditure required for sexual reproduction (Gostinčar et al. 2022a).

Finally, the availability of genomes will facilitate further investigation of the halotolerance of some black yeasts. This should include both data-driven research, such as well-designed and carefully conducted transcriptomic experiments, and hypothesis-driven research that utilises the molecular tools that have become available in the two decades since the first systematic studies of halotolerance in these fungi. Among other tools, CRISPR-Cas9 is increasingly being used for the genetic manipulation of unconventional yeasts, including *A. pullulans* (Zhang et al. 2019; Kreuter et al. 2022), opening up new possibilities for deciphering the complex phenotype of halotolerant black yeasts.
